# Combined effects of body mass index and unhealthy behaviors on disability in older Japanese adults: the Okayama study

**DOI:** 10.7717/peerj.8146

**Published:** 2019-11-29

**Authors:** Yangyang Liu, Toshiharu Mitsuhashi, Michiyo Yamakawa, Megumi Sasai, Toshihide Tsuda, Hiroyuki Doi, Jun Hamada

**Affiliations:** 1 Department of Epidemiology, Graduate School of Medicine, Dentistry and Pharmaceutical Sciences, Okayama University, Okayama, Japan; 2 Center for Innovative Clinical Medicine, Okayama University Hospital, Okayama University, Okayama, Japan; 3 Department of Epidemiology and Preventive Medicine, Graduate School of Medicine, Gifu University, Gifu, Japan; 4 Sri Lanka Office, Japan International Cooperation Agency, Colombo, Sri Lanka; 5 Department of Human Ecology, Graduate School of Environmental and Life Science, Okayama University, Okayama, Japan; 6 Department of Health Economics and Policy, Graduate School of Medicine, Dentistry and Pharmaceutical Sciences, Okayama University, Okayama, Japan

**Keywords:** Elderly, Disability, Body mass index, Long-term care insurance, Unhealthy behaviors

## Abstract

**Background:**

Body mass index (BMI) is a significant predictor of functional disability in older adults. However, when evaluated, the association between BMI and incident functional disability, considering behaviors only as covariates or not, may not be appropriate. The primary purpose of the study was to investigate the combined effects of BMI and unhealthy behaviors on the risk of incident functional disability.

**Methods:**

This was a retrospective cohort study that took place in Okayama City, Japan. Data on BMI and unhealthy behaviors were obtained using the health check-up questionnaire. The certification of Long-Term Care Insurance was used to measure functional disability. Cox proportional hazard models were used; adjusted hazard ratios (HRs) with 95% confidence interval (CI) were calculated for incidence of functional disability across categories of BMI and number of unhealthy behaviors.

**Results:**

The relationship between BMI and incident functional disability was U-shaped (HR = 1.18, 95% CI [1.11–1.25], among the underweight range; and 1.26 [1.19–1.34] among the obesity range), and its risk was significantly higher within the normal-to-overweight range of BMI values with co-occurring unhealthy behaviors (with normal weight range and one, 1.17 [1.01–1.21]; two, 1.29 [1.18–1.41]; and three or four unhealthy behaviors 1.38 [1.24–1.54]; as well as among overweight range and one, 1.16 [1.05–1.27]; two, 1.26 [1.15–1.38]; and three or four unhealthy behaviors, 1.47 [1.31–1.64]). In each BMI category, the risk of incident functional disability increased with increasing number of unhealthy behaviors (*p* < 0.05 for linear tread), with the highest risk (1.87 [1.58–2.20]) occurring in combination with at least three unhealthy behaviors with BMI ≥ 27.5, for both sexes (2.20 [1.64–2.92] in men and 1.66 [1.35–2.04] in women).

**Conclusion:**

It is necessary to consider the combined effects of BMI and behaviors on incident functional disability. Furthermore, interventions targeting multiple behaviors should be considered as such interventions may offer greater benefits than simple interventions.

## Introduction

Over the past 40 years, the number of Japanese aged 65 years or older has increased by a quarter of the total population (Nomura et al., 2017). Both life and healthy life expectancies are increasing, but the disparity between them has widened (Ishii, Ogawa & Akishita, 2015), contributing to the increase in incident functional disability in older adults. Functional disability is now thought to have an enormous effect on hospitalization, institutionalization, and death, leading to adverse effects on the Japanese economy (Tsubota-Utsugi et al., 2011). Therefore, it is necessary to investigate the risk factors of incident functional disability to implement health policies aimed at prolonging healthy life expectancy.

Body mass index (BMI) is a significant predictor of functional disability in older adults (Stuck et al., 1999). Some studies have shown the association between BMI and incident functional disability as a J- or U-shaped curve (Akune et al., 2014; Gadalla, 2010; Larrieu et al., 2004; Yang et al., 2014; Zhang et al., 2016), indicating that the participants who are underweight or obese are both at a higher risk of disability than those with normal BMI. Several studies have also suggested that unhealthy behaviors, such as smoking, heavy or no alcohol consumption, physical inactivity, and unhealthy eating habits, are associated with a greater risk of incident functional disability (Artaud et al., 2013; Sulander et al., 2005). Meanwhile, unhealthy behaviors have also been associated with BMI values. For example, an increased number of unhealthy behaviors can increase the prevalence of obesity (Harrington et al., 2010), and low BMI usually indicates a state of undernutrition (Bahat et al., 2015).

Several studies have considered some behaviors as covariates when the association between BMI and incident functional disability was evaluated. One particular study suggested that a significant interaction existed between BMI and behaviors (Veronese et al., 2016). The effects demonstrated for BMI and unhealthy behaviors may have a synergistic effect on functional disability by increasing the risk of chronic disease when they are considered together. Therefore, considering behaviors only as covariates may not be appropriate. In addition, as unhealthy behaviors tend to cluster in participants, such that those with one unhealthy behavior are more likely to have others (Poortinga, 2007), it might be important to consider prevalent unhealthy behaviors together. Meanwhile, previous studies suggested that BMI is related to physical function, depending on sex (Friedmann, Elasy & Jensen, 2001), with differences in behavioral patterns between men and women (Brandts & Van Den Brandt, 2018; Wardzala et al., 2018). Therefore, it would be better to examine the effects separately by sex. Furthermore, these studies had some limitations, such as inappropriate BMI cut-off points, lack of a longitudinal study design, small sample size, and short follow-up period.

To address these gaps, we applied the World Health Organization (WHO) Asia criteria (WHO Expert Consultation, 2004) to categorize BMI and, studied the combined effects of BMI and unhealthy behaviors (including current smoker, alcohol consumption other than light-to-moderate, physical inactivity, and unhealthy eating habits) on the risk of incident functional disability by sex using large sample size data of a study with a long follow-up period in Japan.

## Materials and Methods

### Study participants

The Okayama Study, a longitudinal retrospective cohort study, was conducted in Okayama city, Okayama prefecture, Japan, in 2018. Okayama is a city encompassing a wide socioeconomic and urban–rural distribution. The aim of the Okayama Study was to investigate the relationship between behaviors and certification for Long-term care insurance (LTCI). The present study was part of the Okayama Study. The requirement for informed consent was waived, as this was a retrospective study using anonymized data. The Ethics Committee of the Okayama University Graduate School of Medicine Dentistry and Pharmaceutical Sciences and Okayama University Hospital reviewed and approved the study (approval number K1703-037).

**Figure 1:**
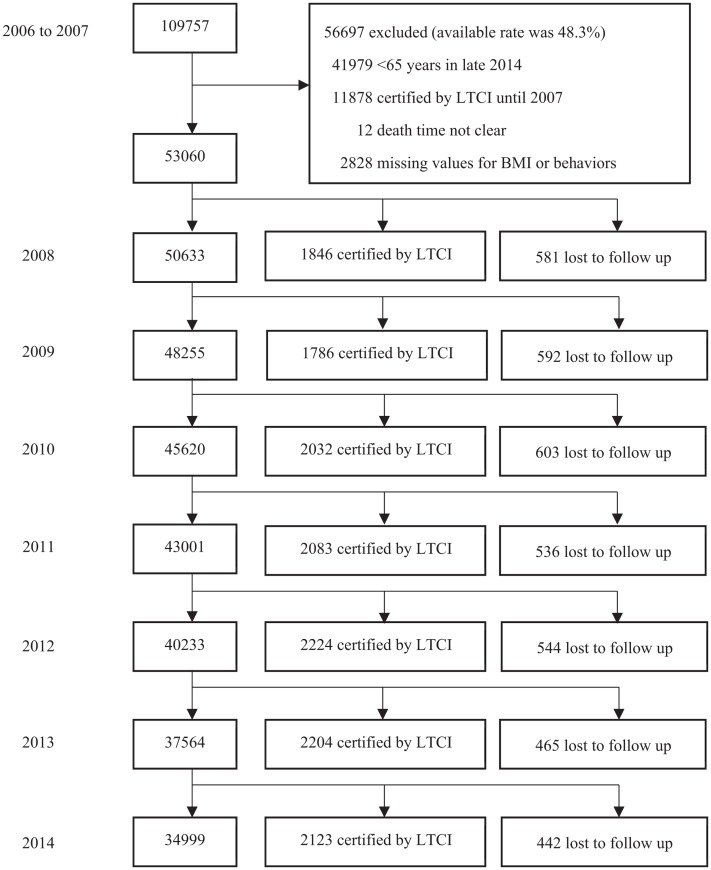
Flowchart of study participants. Abbreviations: BMI, body mass index; LTCI, long-term care insurance. Behaviors include smoking status, physical activity, alcohol consumption, eating habits. Lost to follow up includes death or migration.

Figure 1 shows the flowchart of the study. From 2006 to 2007, the study enrolled 109,757 participants residing in Okayama city who completed the health checkup questionnaire (conducted by Healthy Service of the municipal government of Okayama) for the elderly at a hospital or in the participants’ homes under the assistance of doctors or nurses. We excluded the following persons: 41,979 persons who were still under 65 years in late 2014; 11,878 persons certified by LTCI before follow-up; 12 persons with unknown dates of death; and 2,828 persons with missing values for BMI, smoking, alcohol consumption, physical activity, or eating habits. We did not exclude persons with disease as was done in previous studies (Padwal et al., 2016; Yu et al., 2017; Zhang et al., 2016). Subsequently, 53,060 participants (available rate was 48.3%) were included in our analysis. During the 7-year period, 3,763 participants had not been certified by LTCI until they were lost to follow-up on account of death or migration from the study area. This resulted in a follow-up rate of 92.9%. Among the 309,336 person-years, 14,298 participants were certified by LTCI.

### Assessment of exposure

Based on the measured height and weight, BMI was calculated as weight (kg) divided by the square of height (m^2^). Moreover, the WHO Asia criteria was applied to categorize BMI into four groups: underweight (<18.5 kg/m^2^), normal weight (18.5–23.0 kg/m^2^), overweight (23.0–27.5 kg/m^2^), and obesity (≥27.5 kg/m^2^) (WHO Expert Consultation, 2004). Information on behaviors were obtained from the completed health check-up questionnaire as follows: smoking status was classified as non-smoker (including former smoker) or current smoker (Kvaavik et al., 2010; Tamakoshi et al., 2009). Alcohol consumption was classified as non-drinker, light-to-moderate (equal or less than two units per day), or heavy (more than two units per day) (Minster of Health, Labour & Welfare, 2012). Physical activity was classified as inactivity or regular (more than half an hour of exercise per week for 1 year) (Minster of Health, Labour & Welfare, 2012). Eating habits were classified as healthy or unhealthy (four items, including not controlling salt and calorie intake and intake of few vegetables and calcium, which were associated with several chronic diseases) (Joint WHO/FAO Expert Consultation, 2003; Woo et al., 1998). We considered current smoker, physical inactivity, alcohol consumption other than light-to-moderate, and unhealthy eating habits to be unhealthy behaviors according to previous studies (Artaud et al., 2013; Byun et al., 2010; Chakravarty et al., 2012; Harrington et al., 2010; Sabia et al., 2009; Veronese et al., 2016) and the recommendation of Health Japan 21 (2nd series) from the Ministry of Health, Labor, and Welfare (Minster of Health, Labour & Welfare, 2012).

### Ascertainment of disability

In the present study, certification of LTCI was used as a measure of incident functional disability in older adults—a process that is objectively, fairly, and nationally standardized in Japan (Tsutsui & Muramatsu, 2005). The LTCI is a mandatory national social insurance system, in which everyone aged above 40 years pays premiums, and everyone above 65 years is eligible to apply for certification from the municipal government, based on physical and mental disability as assessed by an objective test (Campbell & Ikegami, 2003). Briefly, a care manager will visit and interview the elderly person at home to assess the physical and mental health status by using several scales developed by the Ministry of Health, Labor, and Welfare. The care managers are licensed professionals with at least 5 years of experience, such as nurses, physicians, social workers, and physical therapists. They have also undertaken a few days of training about the interview. According to the results of the care manager’s investigation and physician’s opinion, municipal certification committee experts for LTCI (who are experienced or are of reputable academic standing in the fields of health, medical treatment, and welfare) determine the certification (eligible or not) and its level. Eligibility includes seven levels, ranging from support level 1 to level 2, and care level 1 to level 5. Support level 1 indicates a limitation in instrumental activities of daily living but independence in basic activities of daily living (Olivares-Tirado & Tamiya, 2013). In previous studies, the certification of LTCI was shown to be well correlated with other assessments of disability. Furthermore, LTCI certification has been applied as a measurement of incident functional disability in older adults by the Japanese government (Olivares-Tirado & Tamiya, 2013); several studies also used LTCI certification to define incident disability and analyzed the association between factors and incident disability in Japan (Akune et al., 2014; Ishii, Ogawa & Akishita, 2015; Nitta et al., 2010; Zhang et al., 2016). If an older adult was determined to be eligible for certification (support level 1 or higher), he/she was regarded as having incident functional disability. We obtained LTCI information from the Okayama City Public Health Center under privacy protection.

### Assessment of covariates

Covariate assessment included age (continuous), sex, current employment (yes/no), self-rated health (good/other than good), and current diseases (yes/no, including heart disease, hypertension, kidney disease, diabetes mellitus, liver disease, anemia, and hyperlipidemia).

### Statistical analysis

The participants contributed person-years from turned age ≥65 years, during the follow-up period, to the date of incident disability, loss to follow-up (death or emigration from Okayama City), or end of the follow-up period (December 31, 2014), whichever came first. The proportional hazards assumption was checked on both Kaplan–Meier curves and log–log curves. No violations of proportionality were observed. Cox proportional hazard models were used to calculate hazard ratio (HR) values for incidence of functional disability with their 95% confidence intervals (CIs) across BMI categories. Three models were used to calculate adjusted HR values as follows: age and sex were the first to be adjusted (model 1); followed by current employment, current diseases, and self-rated health (model 2); and finally, behaviors (model 3). Subgroup analysis by sex were performed to examine for possible heterogeneity.

Then, we evaluated the combined effects of BMI and unhealthy behaviors on the risk of incident functional disability, which was the primary purpose of our study. First, in adjusted models 1 and 2, we calculated the HR values for the incidence of functional disability across the categories of BMI after stratifying the participants by the number of unhealthy behaviors, which defined BMI in the range of 18.5–23.0, as the reference for each number of unhealthy behaviors. Second, in adjusted model 2, we calculated the HR values for incidence of functional disability according to the joint classification of BMI and unhealthy behaviors, which defined both BMI in the range of 18.5–23.0 and no unhealthy behaviors as the reference for all categories. In this step, to test for the linear trends in each BMI range, *p*-value for trend was estimated by entering ordinal numbers of unhealthy behaviors in each group in the Cox regression model. We also ran subgroup analysis by sex in these two steps.

We ran four sensitivity analyses to test the robustness of our results. First, individuals whose disability events occurred within the first 2 years of follow-up were excluded; second, individuals with diseases (including heart disease, hypertension, kidney disease, diabetes mellitus, liver disease, anemia, and hyperlipidemia) were excluded; third, the unhealthy group of eating habits was redefined (more than two items were unhealthy). Finally, weights were assigned to each unhealthy behavior based on the beta coefficients from the multivariable adjusted Cox model with incidence of functional disability.

All statistical analyses were performed by using SPSS version 21 (IBM Corp., Armonk, NY, USA). All *p*-values were two-sided and those less than 0.05 were considered statistically significant.

## Results

### Baseline characteristics

Among 53,060 participants, the mean (SD) age was 71.4 (7.5) years according to the health check-up questionnaire. Table 1 shows the characteristics of the participants according to BMI categories. Participants with higher BMI values were more likely to have a disease. Participants with lower BMI values were more likely to smoke.

**Table 1:** Demographic characteristics of the participants at baseline.

Characteristics	All	BMI (kg/m^2^)				*p*-value
		Underweight	Normal Weight	Overweight	Obesity	
		<18.5	18.5–23.0	23.0–27.5	≥27.5	
No. of participants	53,060	3,853	23,778	20,949	4,480	
Age, years, mean (SD)	71.4 ± 7.5	73.7 ± 8.3	71.2 ± 7.7	71.2 ± 7.0	70.7 ± 7.0	<0.001
Sex, *n* (%)						
Men	18,475 (34.8)	1,170 (30.4)	7,736 (32.5)	8,267 (39.5)	1,302 (29.1)	<0.001
Women	34,585 (65.2)	2,683 (69.6)	16,042 (67.5)	12,682 (60.5)	3,178 (70.9)	
Current employment, *n* (%)	16,072 (30.3)	1,048 (27.2)	7,138 (30.0)	6,510 (31.1)	1,376 (30.7)	<0.001
Current diseases, *n* (%)	30,610 (57.7)	1,906 (49.5)	12,593 (53.0)	12,961 (61.9)	3,150 (70.3)	<0.001
Self-rated health, good, *n* (%)	24,841 (46.8)	1,474 (38.3)	11,097 (46.7)	10,210 (48.7)	2,060 (46.0)	<0.001
Smoking status, *n* (%)						
Non-smoker	47,439 (89.4)	3,297 (85.6)	21,155 (89.0)	18,849 (90.0)	4,138 (92.4)	<0.001
Current smoker	5,621 (10.6)	556 (14.4)	2,623 (11.0)	2,100 (10.0)	342 (7.6)	
Physical activity, *n* (%)						
Inactivity	28,283 (53.3)	2,254 (58.5)	12,258 (51.6)	11,060 (52.8)	2,711 (60.5)	<0.001
Regular physical activity	24,777 (46.7)	1,599 (41.5)	11,520 (48.4)	9,889 (47.2)	1,796 (39.5)	
Alcohol consumption, *n* (%)						
Non-drinker	34,111 (64.3)	2,746 (71.3)	15,436 (64.9)	12,893 (61.5)	3,036 (67.8)	<0.001
Light-to-moderate	15,381 (29.0)	927 (24.1)	6,931 (29.1)	6,393 (30.5)	1,130 (25.2)	
Heavy	3,568 (6.7)	180 (4.7)	1,411 (5.9)	1,663 (7.9)	314 (7.0)	
Eating habits, *n* (%)						
Healthy	44,138 (83.2)	3,113 (80.8)	19,910 (83.7)	17,436 (83.2)	3,679 (82.1)	0.001
Unhealthy	8,922 (16.8)	740 (19.2)	3,868 (16.3)	3,513 (16.8)	801 (17.9)	
No. of unhealthy behaviors, *n* (%)						
0	6,199 (11.7)	324 (8.4)	2,914 (12.3)	2,565 (12.2)	396 (8.8)	<0.001
1	20,144 (38.0)	1,298 (33.7)	9,197 (38.7)	8,095 (38.6)	1,554 (34.7)	
2	20,471 (38.6)	1,605 (41.7)	8,912 (37.5)	7,971 (38.0)	1,983 (44.3)	
3 or 4	6,246 (11.8)	626 (16.2)	2,755 (11.6)	2,318 (11.1)	547 (12.2)	

**Notes:**

BMI, body mass index; SD, standard deviation.

*p*-values obtained by using analysis of variance (ANOVA) for continuous variables and the chi-squared test for categorical variables.

### Association between BMI and incident functional disability

Table 2 shows the association between BMI and incident functional disability, separately. After multivariate adjustment for potential confounders, the results showed significantly higher HR values (95% CI) in the final model: 1.18 [1.11–1.25] overall, and in men (1.27 [1.14–1.40]) and women (1.14 [1.06–1.22]) among the underweight; as well as 1.26 [1.19–1.34] overall, and in men (1.15 [1.02–1.29]) and women (1.30 [1.22–1.40]) among obese participants. However, no significant association was observed in the overweight participants for both sexes.

**Table 2:** Hazard ratios for incidence of functional disability associated with BMI.

	BMI (kg/m^2^)			
	Underweight	Normal weight	Overweight	Obesity
	<18.5	18.5–23.0	23.0–27.5	≥27.5
No. of participants	3,853	23,778	20,949	4,480
Person-years	19,889.0	138,994.0	124,486.5	25,966.0
No. of events	1,411	6,173	5,356	1,358
Crude HR (95% CI)	1.59 [1.50–1.69]	1.00 (reference)	0.97 [0.93–1.01]	1.18 [1.11–1.25]
Adjusted Model 1^a^	1.19 [1.12–1.27]	1.00 (reference)	1.01 [0.98–1.05]	1.29 [1.22–1.37]
Adjusted Model 2^b^	1.20 [1.13–1.27]	1.00 (reference)	1.00 [0.98–1.04]	1.27 [1.20–1.34]
Adjusted Model 3^c^	1.18 [1.11–1.25]	1.00 (reference)	1.01 [0.97–1.04]	1.26 [1.19–1.34]
Men^d^	1.27 [1.14–1.40]	1.00 (reference)	0.95 [0.89–1.02]	1.15 [1.02–1.29]
Women^e^	1.14 [1.06–1.22]	1.00 (reference)	1.04 [0.99–1.09]	1.30 [1.22–1.40]

**Notes:**

BMI, body mass index; PY, person year; HR, hazard radio; CI, confidence interval.

a Adjusted age and sex.

b Adjusted model 1 plus current employment (yes or no), current diseases (yes or no), self-rated health (good or other than good).

c Adjusted model 2 plus smoking status (non-smoker or current smoker), physical activity (inactivity or regular), alcohol consumption (non-drinker, light-to-moderate, or heavy), eating habits (healthy or unhealthy).

d Adjusted for the same covariates in Model 3 without sex.

e Adjusted for the same covariates in Model 3 without sex.

### Combined effects of BMI and unhealthy behaviors on the risk of incident functional disability by the number of unhealthy behaviors

Table 3 shows the combined effects of BMI and unhealthy behaviors on the risk of incident functional disability by the number of unhealthy behaviors, defined only by BMI range 18.5–23.0, as the reference for each number of unhealthy behaviors. After multivariate adjustment for potential confounders, among participants with no unhealthy behaviors, only the HR value (95% CI) for underweight men was significantly high at 1.47 [1.09–1.97]. Of participants with more than one unhealthy behavior, there were significantly higher HR values (95% CI): among the underweight, for one, 1.17 [1.05–1.30]; two, 1.16 [1.06–1.27]; and three or four, 1.24 [1.08–1.43] unhealthy behaviors. Among the obese, for one, 1.18 [1.63–1.31]; two, 1.29 [1.19–1.41]; and three or four, 1.33 [1.14–1.56] unhealthy behaviors. In the subgroup analyses, we found significantly higher HR values (95% CI) for underweight men for one, 1.36 [1.13–1.63] and for three or four, 1.31 [1.06–1.63] unhealthy behaviors. Significantly higher HR values (95% CI) were also observed in women for two unhealthy behaviors among the underweight (1.17 [1.06–1.30]). Among the obese, women with one, 1.27 [1.12–1.44]; two, 1.32 [1.20–1.45]; and three or four, 1.37 [1.13–1.66] unhealthy behaviors also had significantly higher HR values.

**Table 3:** Hazard ratios for incidence of functional disability associated with BMI by number of unhealthy behaviors.

No. of unhealthy behaviors^a^	BMI (kg/m^2^)			
	Underweight	Normal weight	Overweight	Obesity
	<18.5	18.5–23.0	23.0–27.5	≥27.5
None				
No. of events/PYs	93/1,853.0	563/17,924.0	443/16,053.0	84/2,434.5
Crude HR (95% CI)	1.60 [1.29–2.00]	1.00 (reference)	0.88 [0.78–1.00]	1.10 [0.87–1.38]
Adjusted Model 1^b^	1.27 [1.02–1.58]	1.00 (reference)	0.90 [0.80–1.02]	1.19 [0.95–1.50]
Adjusted Model 2^c^	1.22 [0.98–1.53]	1.00 (reference)	0.90 [0.80–1.02]	1.15 [0.91–1.45]
Men^d^	1.47 [1.09–1.97]	1.00 (reference)	0.91 [0.78–1.08]	1.27 [0.91–1.75]
Women^e^	1.01 [0.72–1.42]	1.00 (reference)	0.90 [0.75–1.09]	1.04 [0.75–1.45]
One				
No. of events/PYs	435/7,009.0	2194/54,953.5	1991/48,618.5	406/9,265
Crude HR (95% CI)	1.55 [1.40–1.72]	1.00 (reference)	1.03 [0.97–1.09]	1.10 [0.99–1.22]
Adjusted Model 1^b^	1.16 [1.05–1.29]	1.00 (reference)	1.07 [1.00–1.13]	1.20 [1.08–1.34]
Adjusted Model 2^c^	1.17 [1.05–1.30]	1.00 (reference)	1.05 [0.99–1.12]	1.18 [1.06–1.31]
Men^d^	1.36 [1.13–1.63]	1.00 (reference)	0.98 [0.88–1.09]	0.97 [0.79–1.20]
Women^e^	1.10 [0.97–1.25]	1.00 (reference)	1.08 [1.00–1.17]	1.27 [1.12–1.44]
Two				
No. of events/PYs	625/8,149.0	2607/50,808.0	2244/46,614.0	676/11,236.5
Crude HR (95% CI)	1.49 [1.36–1.62]	1.00 (reference)	0.94 [0.89–1.00]	1.17 [1.08–1.27]
Adjusted Model 1^b^	1.16 [1.06–1.26]	1.00 (reference)	0.99 [0.93–1.04]	1.32 [1.21–1.43]
Adjusted Model 2^c^	1.16 [1.06–1.27]	1.00 (reference)	0.98 [0.92–1.03]	1.29 [1.19–1.41]
Men^d^	1.15 [0.97–1.36]	1.00 (reference)	0.92 [0.82–1.03]	1.20 [0.99–1.45]
Women^e^	1.17 [1.06–1.30]	1.00 (reference)	1.00 [0.93–1.07]	1.32 [1.20–1.45]
Three or four				
No. of events/PYs	258/2,878.5	809/15,308.5	678/13,201.0	192/3,030.0
Crude HR (95% CI)	1.68 [1.46–1.93]	1.00 (reference)	0.97 [0.88–1.08]	1.20 [1.02–1.40]
Adjusted Model 1^b^	1.22 [1.06–1.40]	1.00 (reference)	1.06 [0.96–1.17]	1.37 [1.17–1.60]
Adjusted Model 2^c^	1.24 [1.08–1.43]	1.00 (reference)	1.04 [0.94–1.16]	1.33 [1.14–1.56]
Men^d^	1.31 [1.06–1.63]	1.00 (reference)	0.97 [0.83–1.13]	1.28 [0.97–1.70]
Women^e^	1.19 [0.99–1.43]	1.00 (reference)	1.10 [0.96–1.26]	1.37 [1.13–1.66]

**Notes:**

BMI, body mass index; PY, person year; HR, hazard radio; CI, confidence interval.

a Including current smoker, physical inactivity, alcohol consumption other than light-to-moderate, unhealthy eating habits.

b Adjusted age and sex.

c Adjusted model 1 plus current employment (yes or no), current diseases (yes or no), self-rated health (good or not good).

d Adjusted for the same covariates in Model 2 without sex.

e Adjusted for the same covariates in Model 2 without sex.

### Combined effects of BMI and unhealthy behaviors on the risk of incident functional disability according to the joint classification of BMI and unhealthy behaviors

**Figure 2:**
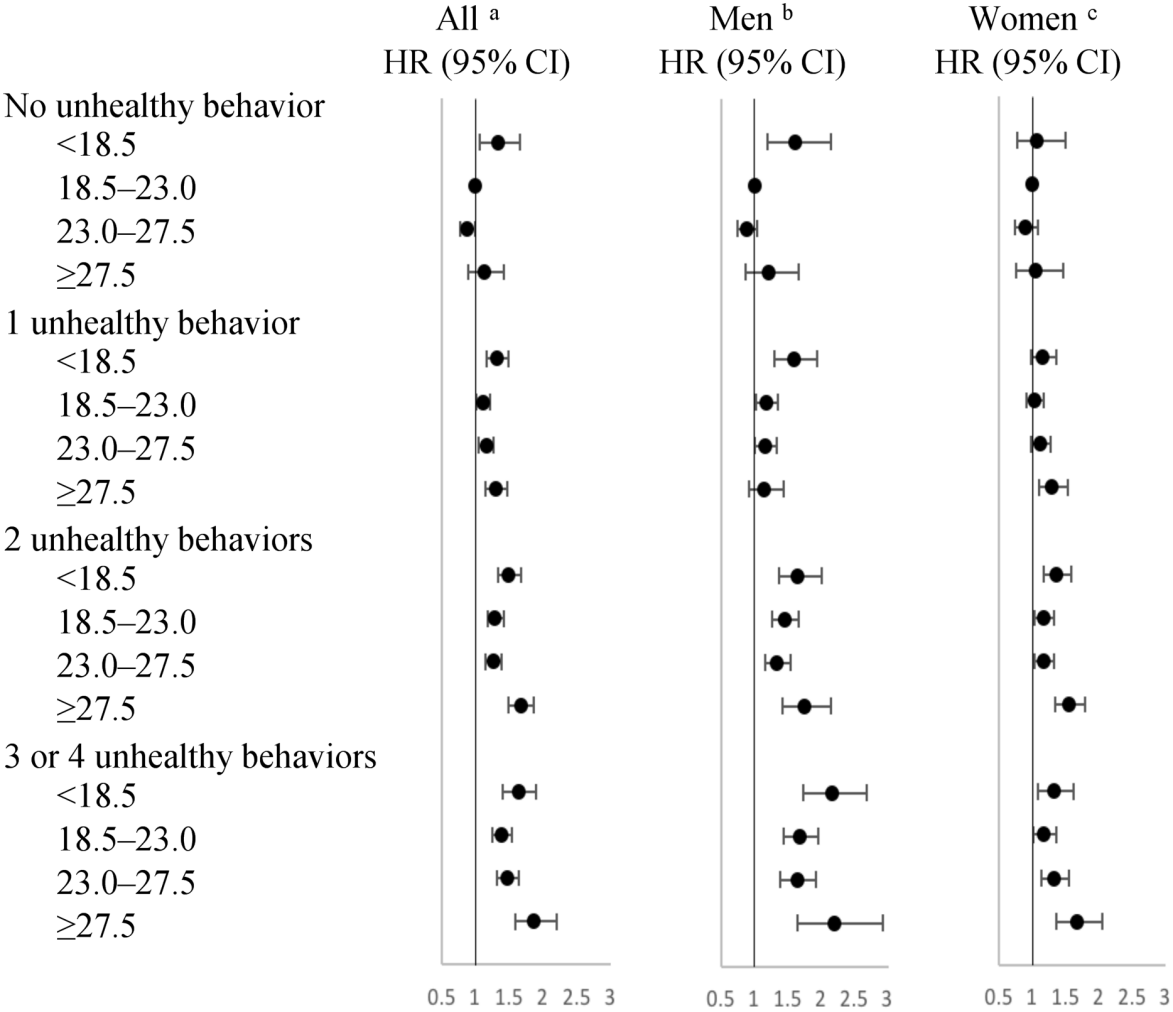
Hazard ratios for incidence of functional disability according to the joint classification of BMI and unhealthy behaviors. Abbreviations: BMI, body mass index; HR, hazard radio; CI, confidence interval. Reference category: both BMI in the range of 18.5–23.0 and no unhealthy behavior. BMI values include four ranges (kg/m^2^): <18.5; 18.5–23.0; 23.0–27.5; ≥27.5. Unhealthy behaviors included current smoker, physical inactivity, alcohol consumption other than light-to-moderate, unhealthy eating habits. ^a^Adjusted for age, sex, current employment (yes or no), current diseases (yes or no), self-rated health (good or other than good); ^b^Adjusted for the same covariates in Model 2 without sex; ^c^Adjusted for the same covariates in Model 2 without sex.

Figure 2 (Table S1) shows the combined effects of BMI and unhealthy behaviors on the risk of incident functional disability according to the joint classification of BMI and unhealthy behaviors, which defined both BMI in the range of 18.5–23.0 and no unhealthy behaviors as the reference. Even when the BMI value was within the normal-to-overweight range, the HR value (95% CI) was still significantly higher in the presence of unhealthy behaviors. These were 1.17 [1.01–1.21] overall, 1.18 [1.03–1.35] in men with one; 1.29 [1.18–1.41] overall, 1.45 [1.26–1.66] in men and 1.17 [1.03–1.33] in women with two; and 1.38 [1.24–1.54] overall, 1.67 [1.43–1.95] in men and 1.17 [1.01–1.36] in women with three or four unhealthy behaviors among normal weight participants. In overweight participants, these were 1.16 [1.05–1.27] overall, 1.16 [1.01–1.33] in men with one; 1.26 [1.15–1.38] overall, 1.34 [1.17–1.54] in men and 1.17 [1.02–1.33] in women with two; and 1.47 [1.31–1.64] overall, 1.64 [1.39–1.93] in men and 1.32 [1.13–1.54] in women with three or four unhealthy behaviors. In each BMI category, the risk of incident functional disability was graded to be increasing (*p* < 0.05 for linear trend) among participants with one, two, three, or four unhealthy behaviors. This linear trend was also significant by sex (*p* < 0.05 for linear trend), and the highest significant HR value (95% CI) in the entire study participants was 1.87 [1.58–2.20] overall, 2.20 [1.64–2.92] in men and 1.66 [1.35–2.04] in women, in combination with both at least three unhealthy behaviors and BMI ≥ 27.5.

### Sensitivity analyses

The results of the sensitivity analyses excluding participants whose disability event occurred within the first 2 years of follow-up (Fig. S1); the results excluding participants with diseases (Fig. S2); the results redefining the unhealthy eating habits group (Fig. S3); and the results assigning weights to each unhealthy behavior (Fig. S4), were similar to those of the main analysis.

## Discussion

In the present study of 53,060 Japanese older adults, during 7 years of follow-up and after adjustment for potential confounders, when we examined the association between BMI and incident functional disability separately, the results indicated a significantly higher risk of incident functional disability among underweight and obese participants. However, when we evaluated the combined effects of BMI and unhealthy behaviors on the risk of incident functional disability by the number of unhealthy behaviors, the results showed that the risk among several categories of underweight and obese participants became non-significant, while the trend of U-shaped curves remained. Furthermore, when we evaluated the combined effects of BMI and unhealthy behaviors on the risk of incident functional disability according to the joint classification of BMI and unhealthy behaviors, the results suggested that even when BMI value was within the normal-to-overweight range, the risk might also be significantly higher in the presence of unhealthy behaviors. In each BMI category, the risk of disability increased progressively with the increasing number of unhealthy behaviors, and the highest risk was in combination with at least three unhealthy behaviors and BMI ≥ 27.5 for both sexes.

Many epidemiological studies had shown a U-shaped relationship between BMI and risk of incident function disability; however, they did not adjust for behavioral factors (Akune et al., 2014; Gadalla, 2010), or only adjusted for them as covariates when considering behavioral factors (Larrieu et al., 2004; Yang et al., 2014; Zhang et al., 2016). Using Asian criteria of BMI cut-off points, after adjustment for more potential behaviors, including smoking status, alcohol consumption, physical activity, and eating habits, our study confirmed that an increased risk of incident functional disability was associated with BMI < 18.5 and BMI ≥ 27.5, for both sexes.

In the combined effects by the number of unhealthy behaviors, the risk among several categories of underweight or obesity became non-significant, and the change was not entirely the same between men and women. This might be explained by the fact that a significant interaction existed between BMI and unhealthy behaviors, and the effects were different between men and women. This might be due to differences in behavioral patterns, body composition, and susceptibility to unhealthy behaviors (National Institute on Alcohol Abuse & Alcoholism, 1999; Brandts & Van Den Brandt, 2018; Taki et al., 2011; Wardzala et al., 2018).

Furthermore, in the combined effects according to the joint classification of BMI and unhealthy behaviors, the risk was also significantly higher in the presence of unhealthy behaviors even when the BMI value was in the normal-to-overweight range. This might be explained by the fact that those who were within the normal-overweight range, may be driven by other reasons that can increase the risk of incident functional disability, such as chronic disease and neurodegenerative diseases that are associated with unhealthy behaviors (Booth, Roberts & Laye, 2012; Chiolero et al., 2008; Weyerer et al., 2011; Woo et al., 1998). In each BMI category, the risk increased progressively with increasing number of unhealthy behaviors. Several studies have examined the combined effects of behaviors on health outcome (Byun et al., 2010; Chakravarty et al., 2012; Harrington et al., 2010; Veronese et al., 2016), and their findings supported our present findings (Artaud et al., 2013; Loef & Walach, 2012). However, they did not investigate the unhealthy behaviors by sex. Our results also suggested that the cumulative effects by the number of unhealthy behaviors existed in both sexes (*p* < 0.05 for linear trend), and although the effect size was different, the trend also existed for both men and women.

The strengths of this study include its large sample size, the long follow-up period with large numbers of incident functional disabilities, high follow-up rate, use of Asian criteria of BMI cut-off points, and consideration of many confounding factors. In addition, the present study evaluated the combined effects of BMI and unhealthy behaviors on the risk of incident functional disability by sex rather than by considering only some unhealthy behaviors as covariates or not being considered at all. Our study also has several potential limitations. First, not all participants who completed the health checkup questionnaire were included in our analysis; thus, the study may not be free of selection bias. However, a previous study suggested that the proportion of missing data of less than 10% might not influence the results (Hancock, Mueller & Stapleton, 2010). Second, behaviors were assessed by self-report; thus, recall bias and misclassifications cannot be ruled out. However, these would be non-differential or show strong association between retrospective self-reported and subjective measures on behaviors (Middleton et al., 2011; Stampfer et al., 2005). Third, BMI and behaviors were assessed at baseline, and we were unable to evaluate changes over the follow-up period for individuals under 65 years. However, BMI and behaviors have been shown to be relatively stable over time in older adults (Artaud et al., 2013). Fourth, the grouping of alcohol consumption may be controversial. Several previous studies suggested that no safe level of alcohol consumption existed; therefore, the results need to be considered carefully before recommending non-drinkers to drink. Fifth, the process of assigning simple values to behaviors was crude. However, the results of the sensitivity analyses, in which a weight was assigned to the values of behaviors, were consistent with our main analysis. In addition, a previous study has suggested that no evidence exists that a particular combination of unhealthy behaviors drives the association (Sabia et al., 2009). Sixth, not all candidates applied for LTCI certification; thus, the present study may not be free of information bias. However, this would be a non-differential misclassification of outcome status, which will not invert the direction of the relationship (Szklo & Nieto, 2014). Finally, although the models adjusted for numerous potential covariates, the results might still be distorted by residual confounders, such as socioeconomic factors, which could not be addressed in our study. However, in terms of socioeconomic factors, the income gap is relatively small among older Japanese individuals (Kondo et al., 2012), and after adjusting for current employment, the results remained unchanged.

## Conclusions

According to the joint classification of BMI and unhealthy behaviors, the risk of incident functional disability was significantly higher within the normal-to-overweight range of BMI value in the presence of unhealthy behaviors. Furthermore, the risk increased progressively with increasing number of unhealthy behaviors in each BMI category, and the highest risk was in the combination with at least three unhealthy behaviors and BMI ≥ 27.5, for both sexes. It was therefore necessary to consider the combined effects of BMI and behaviors on the risk of incident functional disability, and that interventions targeting multiple behaviors may offer greater benefits than simple interventions. Moreover, we used the certification of LTCI as a measure of incident functional disability; thus, our results could provide new evidence regarding the development of appropriate interventions for preventing disability and delaying the certifications for LTCI in older Japanese adults for use by policy makers as well as by clinicians. In future studies, it is necessary to investigate the mechanisms of disability with respect to these risk factors.

## Supplemental Information

10.7717/peerj.8146/supp-1 Supplemental Information 1 Raw data. The variates of exposures (BMI and unhealthy behaviors), outcome (disability), and covariates (age, sex, current employment, current diseases, and self-rated health). These variates were used for statistical analysis to study the combined effect of BMI and unhealthy behaviors on the risk of incident disability.

10.7717/peerj.8146/supp-2 Supplemental Information 2 Hazard ratio for incidence of functional disability according to the joint classification of BMI and unhealthy behaviors. Abbreviations: BMI, body mass index; HR, hazard radio; CI, confidence interval.
Reference category: both BMI in the range of 18.5–23.0 (normal weight) and no unhealthy behavior.
BMI values include four ranges: underweight (<18.5 kg/m^2^), normal weight (18.5–23.0 kg/m^2^), overweight (23.0–27.5 kg/m^2^), and obesity (≥27.5 kg/m^2^).
Unhealthy behaviors included current smoker, physical inactivity, alcohol consumption other than light-to-moderate, unhealthy eating habits.
^a^Adjusted model 2, including age, sex, current employment (yes or no), current disease (yes or no), self-rated health (good or other than good); ^b^Adjusted for the same covariates in model 2 without sex; ^c^Adjusted for the same covariates in model 2 without sex.

10.7717/peerj.8146/supp-3 Supplemental Information 3 Hazard ratios for incidence of functional disability according to the joint classification of BMI and unhealthy behaviors with excluding participants whose disability event occurred in the first 2 years of follow-up. Abbreviations: BMI, body mass index; HR, hazard radio; CI, confidence interval.
Reference category: Both BMI in the range of 18.5–23.0 and no unhealthy behavior.
BMI values include four ranges (kg/m^2^): <18.5; 18.5–23.0; 23.0–27.5; ≥27.5.
Unhealthy behaviors included current smoker, physical inactivity, alcohol consumption other than light-to-moderate, unhealthy eating habits.
^a^Adjusted for age, sex, current employment (yes or no), current diseases (yes or no), self-rated health (good or other than good); ^b^Adjusted for the same covariates in Model 2 without sex; ^c^Adjusted for the same covariates in Model 2 without sex.

10.7717/peerj.8146/supp-4 Supplemental Information 4 Hazard ratios for incidence of functional disability according to the joint classification of BMI and unhealthy behaviors with excluding participants with diseases. Abbreviations: BMI, body mass index; HR, hazard radio; CI, confidence interval.
Reference category: both BMI in the range of 18.5–23.0 and no unhealthy behavior; BMI values include four ranges (kg/m^2^): <18.5; 18.5–23.0; 23.0–27.5; ≥27.5.
Unhealthy behaviors included current smoker, physical inactivity, alcohol consumption other than light-to-moderate, unhealthy eating habits.
Diseases include cardiovascular disease, hypertension, kidney disease, diabetes mellitus, liver disease, anemia, and hyperlipidemia.
^a^Adjusted for age, sex, current employment (yes or no), current diseases (yes or no), self-rated health (good or other than good); ^b^Adjusted for the same covariates in Model 2 without sex.
^c^Adjusted for the same covariates in Model 2 without sex.

10.7717/peerj.8146/supp-5 Supplemental Information 5 Hazard ratios for incidence of functional disability according to the joint classification of BMI and unhealthy behaviors with redefining unhealthy eating habits groups. Abbreviations: BMI, body mass index; HR, hazard radio; CI, confidence interval.
Reference category: both BMI in the range of 18.5–23.0 and no unhealthy behavior.
BMI values include four ranges (kg/m^2^): <18.5; 18.5–23.0; 23.0–27.5; ≥7.5.
Unhealthy behaviors included current smoker, physical inactivity, alcohol consumption other than light-to-moderate, unhealthy eating habits.
Defining with more than two unhealthy items as unhealthy eating habits.
^a^Adjusted for age, sex, current employment (yes or no), current diseases (yes or no), self-rated health (good or other than good); ^b^Adjusted for the same covariates in Model 2 without sex; ^c^Adjusted for the same covariates in Model 2 without sex.

10.7717/peerj.8146/supp-6 Supplemental Information 6 Hazard ratios for incidence of functional disability according to the joint classification of BMI and unhealthy behaviors with assigning weights to each unhealthy behavior. Abbreviations: BMI, body mass index; HR, hazard radio; CI, confidence interval.
Reference category: both BMI in the range of 18.5–23.0 and no unhealthy behavior.
BMI values include four ranges (kg/m^2^): <18.5; 18.5–23.0; 23.0–27.5; ≥ 27.5.
Unhealthy behaviors included current smoker, physical inactivity, alcohol consumption other than light-to-moderate, unhealthy eating habits.
Unhealthy behavior score was calculated by the following steps: Firstly, assigned weights to each unhealthy behavior based on the beta-coefficients from the multivariable adjusted cox model with incidence of functional disability as the outcome, secondly summed up the results of unhealthy behavior score multiplied by its weight, divided it by the sum of all beta coefficient values, and then multiplied by four.
^a^Adjusted for age, sex, current employment (yes or no), current diseases (yes or no), self-rated health (good or other than good); ^b^Adjusted for the same covariates in Model 2 without sex; ^c^Adjusted for the same covariates in Model 2 without sex.

## Additional Information and Declarations

### Data Availability

The following information was supplied regarding data availability: The raw data is available in a Supplemental File.
