# Supplementary material for: Combined effects of body mass index and unhealthy behaviors on disability in older Japanese adults: the Okayama study
Source: PeerJ. 2019 Nov 29;7:e8146. doi: 10.7717/peerj.8146 (PMC6886483; doi:10.7717/peerj.8146)
Supplement: Supplemental Information 2 — Abbreviations: BMI, body mass index; HR, hazard radio; CI, confidence interval. Reference category: both BMI in the range of 18.5–23.0 (normal weight) and no unhealthy behavior. BMI values include four ranges: underweight (<18.5 kg/m2), normal weight (18.5–23.0 kg/m2), overweight (23.0–27.5 kg/m2), and obesity (≥27.5 kg/m2). Unhealthy behaviors included current smoker, physical inactivity, alcohol consumption other than light-to-moderate, unhealthy eating habits. aAdjusted model 2, including age, sex, current employment (yes or no), current disease (yes or no), self-rated health (good or other than good); bAdjusted for the same covariates in model 2 without sex; cAdjusted for the same covariates in model 2 without sex. [file peerj-07-8146-s002.docx]

|  | | | | | | | |
| --- | --- | --- | --- | --- | --- | --- | --- |
|  |  | All ^a^ | | Men ^b^ | | Women ^c^ | |
|  |  | HR (95% CI) | | HR (95% CI) | | HR (95% CI) | |
| **No unhealthy behavior** | |  |  |  |  |  |  |
| Underweight | | 1.33 (1.07–1.66) | | 1.60 (1.20–2.14) | | 1.07 (0.77–1.50) | |
| Normal weight | | 1.00 (Reference) | | 1.00 (Reference) | | 1.00 (Reference) | |
| Overweight | | 0.88 (0.77–0.99) | | 0.88 (0.75–1.04) | | 0.89 (0.74–1.08) | |
| Obesity | | 1.13 (0.90–1.42) | | 1.21 (0.87–1.67) | | 1.05 (0.76–1.45) | |
| **1 unhealthy behavior** | |  |  |  | |  | |
| Underweight | | 1.32 (1.16–1.49) | | 1.59 (1.30–1.94) | | 1.15 (0.97–1.36) | |
| Normal weight | | 1.17 (1.01–1.21) | | 1.18 (1.03–1.35) | | 1.02 (0.90–1.17) | |
| Overweight | | 1.16 (1.05–1.27) | | 1.16 (1.01–1.33) | | 1.11 (0.97–1.26) | |
| Obesity | | 1.30 (1.14–1.46) | | 1.14 (0.91–1.43) | | 1.29 (1.10–1.52) | |
| **2 unhealthy behaviors** | |  |  |  | |  | |
| Underweight | | 1.49 (1.33–1.67) | | 1.65 (1.36–2.00) | | 1.36 (1.17–1.58) | |
| Normal weight | | 1.29 (1.18–1.41) | | 1.45 (1.26–1.66) | | 1.17 (1.03–1.33) | |
| Overweight | | 1.26 (1.15–1.38) | | 1.34 (1.17–1.54) | | 1.17 (1.02–1.33) | |
| Obesity | | 1.67 (1.49–1.87) | | 1.74 (1.41–2.14) | | 1.55 (1.34–1.79) | |
| **3 or 4 unhealthy behaviors** | |  |  |  | |  | |
| Underweight | | 1.63 (1.41–1.90) | | 2.15 (1.73–2.68) | | 1.32 (1.08–1.62) | |
| Normal weight | | 1.38 (1.24–1.54) | | 1.67 (1.43–1.95) | | 1.17 (1.01–1.36) | |
| Overweight | | 1.47 (1.31–1.64) | | 1.64 (1.39–1.93) | | 1.32 (1.13–1.54) | |
| Obesity | | 1.87 (1.58–2.20) | | 2.20 (1.64–2.92) | | 1.66 (1.35–2.04) | |
|  | | | | | | | |
