# Supplementary figures and images for: Combined effects of body mass index and unhealthy behaviors on disability in older Japanese adults: the Okayama study
Source: PeerJ. 2019 Nov 29;7:e8146. doi: 10.7717/peerj.8146 (PMC6886483; doi:10.7717/peerj.8146)

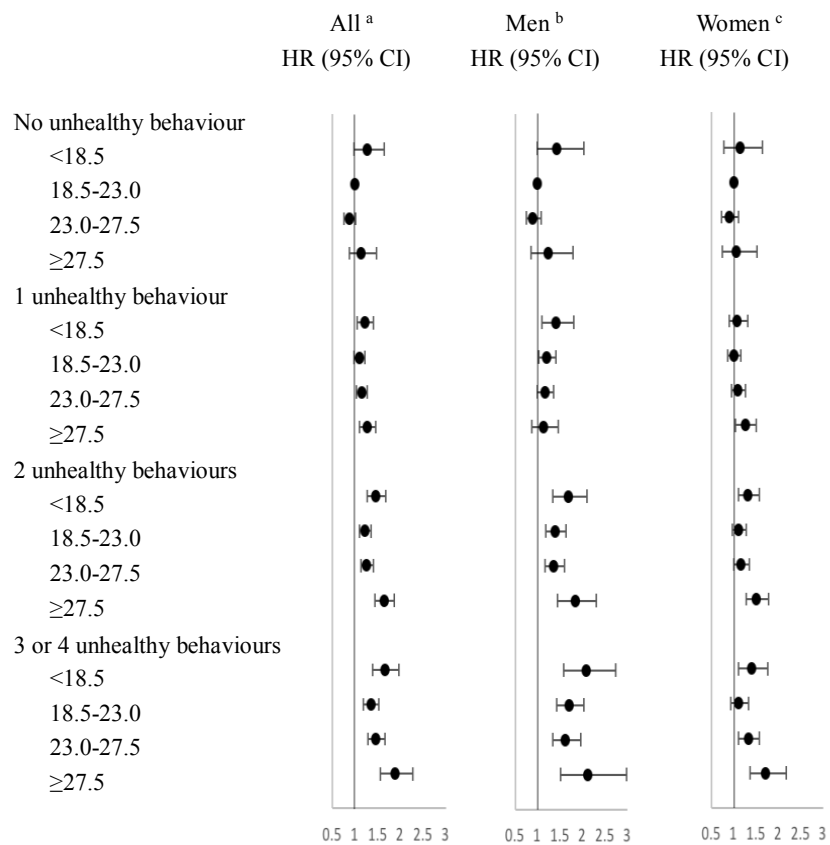

Supplement: Supplemental Information 3 — Abbreviations: BMI, body mass index; HR, hazard radio; CI, confidence interval. Reference category: Both BMI in the range of 18.5–23.0 and no unhealthy behavior. BMI values include four ranges (kg/m2): <18.5; 18.5–23.0; 23.0–27.5; ≥27.5. Unhealthy behaviors included current smoker, physical inactivity, alcohol consumption other than light-to-moderate, unhealthy eating habits. aAdjusted for age, sex, current employment (yes or no), current diseases (yes or no), self-rated health (good or other than good); bAdjusted for the same covariates in Model 2 without sex; cAdjusted for the same covariates in Model 2 without sex. [file peerj-07-8146-s003.pdf]

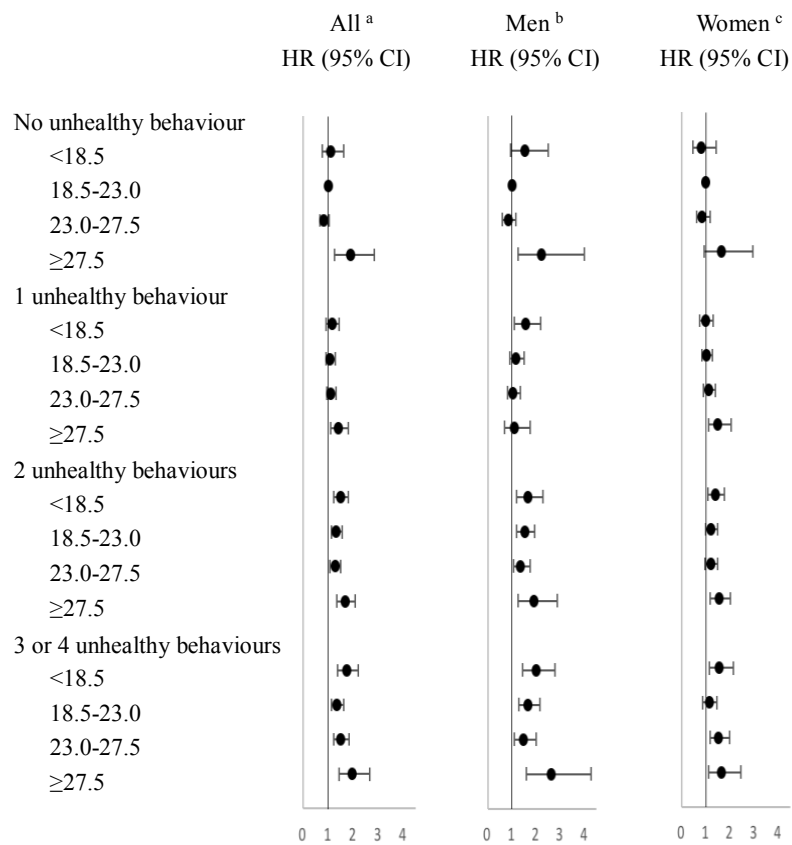

Supplement: Supplemental Information 4 — Abbreviations: BMI, body mass index; HR, hazard radio; CI, confidence interval. Reference category: both BMI in the range of 18.5–23.0 and no unhealthy behavior; BMI values include four ranges (kg/m2): <18.5; 18.5–23.0; 23.0–27.5; ≥27.5. Unhealthy behaviors included current smoker, physical inactivity, alcohol consumption other than light-to-moderate, unhealthy eating habits. Diseases include cardiovascular disease, hypertension, kidney disease, diabetes mellitus, liver disease, anemia, and hyperlipidemia. aAdjusted for age, sex, current employment (yes or no), current diseases (yes or no), self-rated health (good or other than good); bAdjusted for the same covariates in Model 2 without sex. cAdjusted for the same covariates in Model 2 without sex. [file peerj-07-8146-s004.pdf]

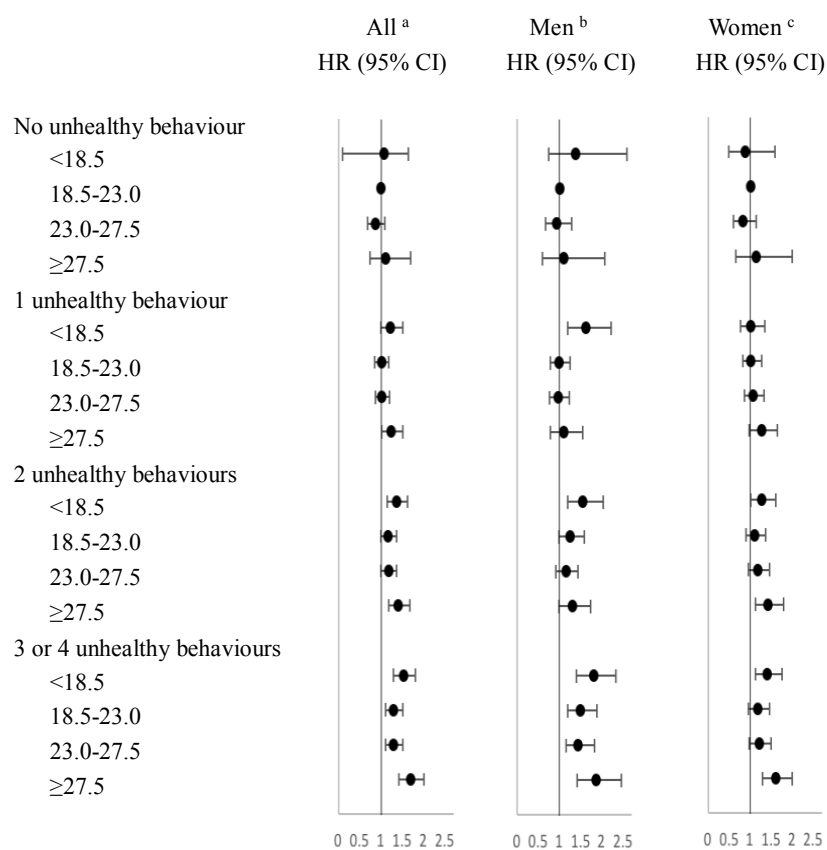

Supplement: Supplemental Information 5 — Abbreviations: BMI, body mass index; HR, hazard radio; CI, confidence interval. Reference category: both BMI in the range of 18.5–23.0 and no unhealthy behavior. BMI values include four ranges (kg/m2): <18.5; 18.5–23.0; 23.0–27.5; ≥7.5. Unhealthy behaviors included current smoker, physical inactivity, alcohol consumption other than light-to-moderate, unhealthy eating habits. Defining with more than two unhealthy items as unhealthy eating habits. aAdjusted for age, sex, current employment (yes or no), current diseases (yes or no), self-rated health (good or other than good); bAdjusted for the same covariates in Model 2 without sex; cAdjusted for the same covariates in Model 2 without sex. [file peerj-07-8146-s005.pdf]

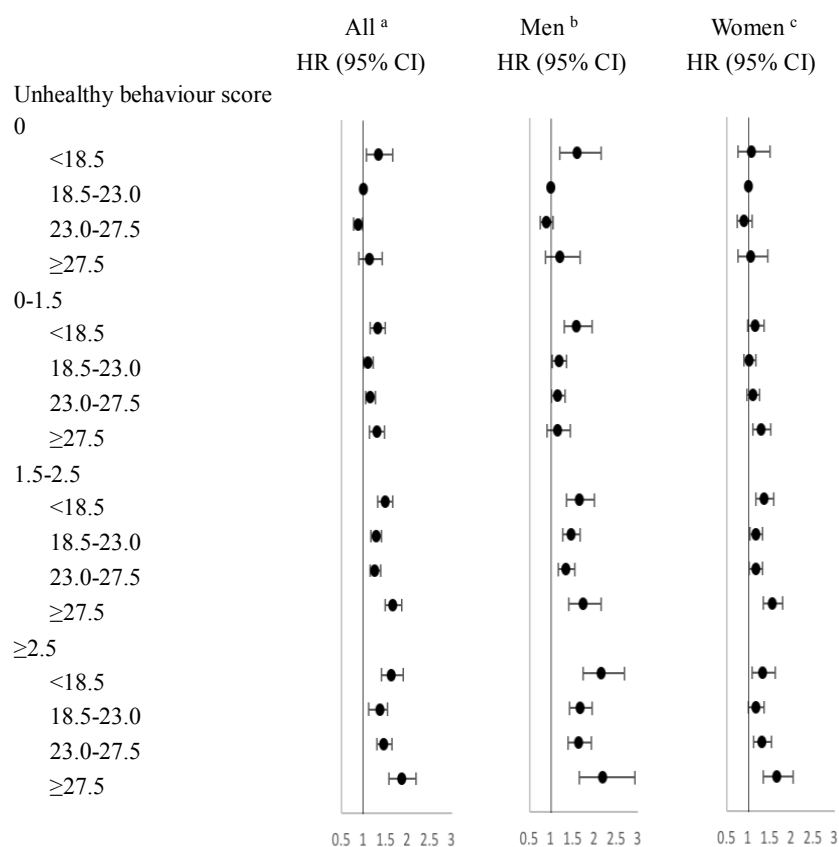

Supplement: Supplemental Information 6 — Abbreviations: BMI, body mass index; HR, hazard radio; CI, confidence interval. Reference category: both BMI in the range of 18.5–23.0 and no unhealthy behavior. BMI values include four ranges (kg/m2): <18.5; 18.5–23.0; 23.0–27.5; ≥ 27.5. Unhealthy behaviors included current smoker, physical inactivity, alcohol consumption other than light-to-moderate, unhealthy eating habits. Unhealthy behavior score was calculated by the following steps: Firstly, assigned weights to each unhealthy behavior based on the beta-coefficients from the multivariable adjusted cox model with incidence of functional disability as the outcome, secondly summed up the results of unhealthy behavior score multiplied by its weight, divided it by the sum of all beta coefficient values, and then multiplied by four. aAdjusted for age, sex, current employment (yes or no), current diseases (yes or no), self-rated health (good or other than good); bAdjusted for the same covariates in Model 2 without sex; cAdjusted for the same covariates in Model 2 without sex. [file peerj-07-8146-s006.pdf]
